# The impact of inflammatory and oxidative stress biomarkers on the sympathetic nervous system in severe coronary atherosclerosis

**DOI:** 10.3389/fcvm.2024.1480925

**Published:** 2024-10-14

**Authors:** Alexandra Maria Boieriu, Cezar Dumitrel Luca, Carmen Daniela Neculoiu, Diana Ţînţ

**Affiliations:** ^1^ Faculty of Medicine, Transilvania University of Brasov, Brasov, Romania; ^2^ Department of Cardiology, Emergency County Hospital, Brasov, Romania; ^3^ Cardiovascular Rehabilitation Hospital Covasna, Covasna, Romania; ^4^ Clinical Laboratory, Emergency County Hospital, Brasov, Romania; ^5^ Department of Cardiology, ICCO Clinics, Brasov, Romania

**Keywords:** inflammation, coronary artery disease, oxidative stress, sympathetic nervous system, coronary artery bypass graft

## Abstract

**Objective:**

The present study aimed at evaluating the association between sympathetic nervous system activation (SNS) and the severity of coronary artery disease (CAD). In addition, we tested the hypothesis that inflammation and oxidative stress influence the SNS activation.

**Methods:**

Adult patients with severe CAD scheduled for coronary artery bypass graft (CABG) surgery were enrolled. SYNTAX I score was calculated based on coronary angiography. Systemic activation of the SNS was estimated through circulating levels of norepinephrine (NE). Plasma levels of pro-inflammatory cytokines (IL 1β, IL 6 and HIF 1α) and oxidative stress molecules (SOD-1 and LOX-1) were obtained prior to surgery.

**Results:**

Circulating NE levels were significantly correlated with the severity of CAD, as assessed by the SYNTAX I score (*p* 0.002; *r* 0.329). Elevated levels of circulating pro-inflammatory markers were significantly correlated with increased NE concentrations (for IL-1β: *p* < 0.001, *r* = 0.49; for IL-6 and NE: *p* = 0.003, *r* = 0.32; for HIF-1α and NE: *p* = 0.049, *r* = 0.21). Additionally, oxidative stress molecules were associated with circulating NE levels (for SOD-1 and NE: *p* = 0.016, *r* = 0.26; for LOX-1 and NE: *p* = 0.004, *r* = 0.31).

**Conclusion:**

In patients with CAD referred for CABG, SNS activation, indicated by plasma NE levels, was correlated with disease severity as assessed by the SYNTAX I score, as well as with markers of inflammation and oxidative stress. This suggests that inflammation, oxidative stress, and SNS activation form an interconnected network, with each component influencing the others. It might be of interest to develop a scoring system including inflammation and oxidative stress markers to identify patients that require a more aggressive approach to lower inflammation, oxidative stress and modulate the sympathetic nervous system. This could be of use especially in the setting of a scheduled intervention -such as CABG surgery.

## 1 Introduction

Inflammation plays a pivotal role in all stages of atherosclerosis, from subclinical endothelial dysfunction to severe atheroma burden and acute cardiovascular events (1). The interleukine-1 (IL-1) family is a major cytokine family (to date 11 members have been discovered; initially comprising only two forms, IL 1 α and IL 1 β) an associated with various cardiovascular diseases (2). Interleukine-6 (IL-6), also an important marker of inflammation, is independently associated with major adverse cardiovascular disease (2, 3). Previous studies have shown that injections of IL 1 and IL 6, pro inflammatory cytokines, cause an activation of the SNS, with release of norepinephrine (NE) from the central as well as the peripheral SNS (4). Hypoxic areas in atheroma plaques lead to the expression of hypoxia-inducible factor 1-alpha (HIF 1 α). HIF 1 α influences cellular functions and cytokine expression inmacrophages, vascular smooth muscle cells and endothelial cells, key elements of the atherosclerotic process (5). Oxidative stress contributes to the development of coronary artery disease (6), lectin-like oxidized low-density lipoprotein receptor-1 (LOX-1), of the scavenger receptors for oxidized low-density lipoprotein cholesterol (ox-LDL)- and superoxide dismutase-1 (SOD 1) being crucial components of the process. Oxidative modifications of low-density lipoprotein to oxidized LDL play an important role in the initiation and progression of atherosclerosis. LOX-1 was identified as the major oxidized LDL receptor in endothelial cells (7). SOD 1 (along with other enzymes in the SOD family) protects cells by scavenging harmful superoxide radicals. SOD is an important marker of lipid peroxidation and of the progression of atherosclerosis correlated with oxidative stress (8).

In ischemic cardiomyopathy aberrant remodeling of the SNS occurs, with effects mainly modulated by NE. Histologic changes in stellate ganglia neurons have been observed resulting in inflammation, glial cell activation, and oxidative stress (9, 10).

Growing evidence suggests that the autonomic nervous system (ANS) plays a crucial role in regulating systemic inflammation, and an imbalance in the ANS may elevate the risk of acute cardiovascular events by promoting inflammation and damaging the endothelium (11).

Clinical studies have previously demonstrated an inverse correlation between SNS activation, assessed through heart rate variability, and chronic low-grade systemic inflammation in patients with stable CAD (12). However, it remains unclear whether SNS activation and inflammation are directly associated with the severity of CAD.

This study aimed to evaluate the association between norepinephrine (NE), as a surrogate marker of sympathetic nervous system (SNS) activation, and the severity of coronary artery disease (CAD), as determined by the SYNTAX score. Additionally, it sought to investigate potential correlations between inflammation, measured by IL-1β, IL-6, and HIF-1α, oxidative stress mediators, such as LOX-1 and SOD-1, and SNS activation, assessed through plasma NE levels, in patients with severe CAD who are candidates for surgical revascularization.

## 2 Methods

### 2.1 Design, study population

This study prospectively included 84 adult patients with severe CAD scheduled for CABG surgery, between January 2020 and June 2021. The study protocol obtained ethical clearance from the Ethical Committee of Transylvania University (registration number 1/2.03.2019) and adhered to the principles outlined in the Helsinki Declaration and the Code for Good Clinical Practice. A written Informed Consent was obtained from all patients.

Patients with acute coronary syndromes, significant valvular disease, severe hepatic or renal failure, recent or active bleeding, coagulation disorders, active malignancy, and inflammatory diseases (including infections and autoimmune disorders) were excluded from the study. Additionally, patients diagnosed with pheochromocytoma or those undergoing psychiatric treatment (such as with serotonin-norepinephrine reuptake inhibitors) were excluded due to the potential impact on norepinephrine (NE) levels. Patients taking alpha-2 blockers were also not included, and alpha agonists were not administered during anesthesia.

At admission, all patients underwent clinical, echocardiographic, and coronary angiography evaluations, and blood samples were collected in accordance with the clinic's protocol. Based on coronary angiogram, the SYNTAX I score was determined using the number of diseased arteries, the location, and the aspect of atherosclerotic plaques (https://syntaxscore.org/).

### 2.2 Measurement of biomarkers

Peripheral venous blood samples were drawn after a minimum of 8 h fasting, 3 days prior CABG. Samples were centrifuged and supernatant was frozen at −80°C until the final measurements. HIF 1 α, IL 1β, IL 6, LOX-1, and NE concentrations were determined by enzyme-linked immunosorbent assay (ELISA) and SOD-1 concentrations were measured using colorimetric determinations, with commercially available kits (Elabscience Biotechnology Inc., Houston, Texas, United States) in accordance with the manufacturer's instructions. All measurements were completed by the same technician who had no access to clinical information.

### 2.3 Statistical analysis

Categorical variables were expressed as *n* (%), normally distributed data and skewed data of continuous variables were expressed as mean ± standard deviation (SD), and median (minimum-maximum), respectively. The normality of continuous variables was tested by Shapiro-Wilk's test. To ascertain distinctions in the analyzed data, 2-tailed Pearson correlation (for variables with normal distribution) and Spearman's correlation (for variables with skewed distribution) were applied. Statistical significance was set at *p* < 0.05. Analysis was performed using Microsoft Excel 2007 and JASP 0.19 software.

## 3 Results

The study population consisted of 84 patients aged 46–88 (mean age: 65), of which 67 were males. The mean body mass index (BMI) was 28.36 kg/m^2^. The documented cardiovascular risk factors included smoking history (20.23%), dyslipidemia (95.23%), hypertension (94.04%), and diabetes mellitus (39.28%). Patients' characteristics and laboratory test findings are presented in Table 1.

**Table 1:** Patients’ characteristics.

Variables	
Age (years) mean ± SD	65 ± 8.09
Male gender *n*, (%)	67 (79.76)
BMI (kg/m^2^) mean ± SD	28.36 ± 4.24
Cardiovascular risk factors *n*, (%)	
Smoking history	17 (20.23)
Dyslipidemia	80 (95.23)
Hypertension	79 (94.04)
Diabetes mellitus	33 (39.28)
SYNTAX score median (min, max)	29.75 (17, 54)
Laboratory data	
IL 1 β (pg/ml) mean ± SD	8.41 ± 1.92
IL 6 (pg/ml) mean ± SD	80.13 ± 10.07
HIF 1 α (pg/ml) median, (min-max)	201.10 (55.54–585.91)
LOX 1 (pg/ml) median, (min-max)	1,191.54 (789.0–2,457.90)
SOD 1 (ng/ml)mean ± SD	1.37 ± 0.23
NE (pg/ml) median, (min-max)	2,964.18 (1,356.89–3,622.62)

BMI, body mass index; SD, standard deviation; IL 1β, interleukine 1β; IL 6, interleukine 6; HIF 1 α, hypoxia-inducible factor 1-alpha; LOX-1, lectin-like oxidized low-density lipoprotein receptor-1; SOD 1, superoxide dismutase-1; NE, norepinephrine.

**Figure 1:**
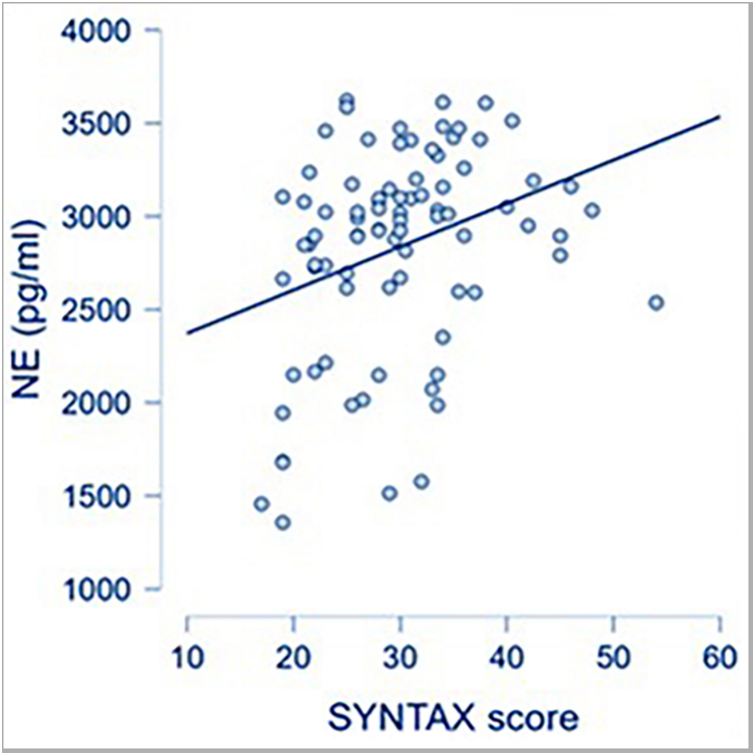
Correlation between SYNTAX score and NE (*p* = 0.002; *r* = 0.329).

CAD severity, as reflected by a high SYNTAX score, was associated with increased systemic levels of NE, used as a surrogate for SNS activation (Figure 1).

**Figure 2:**
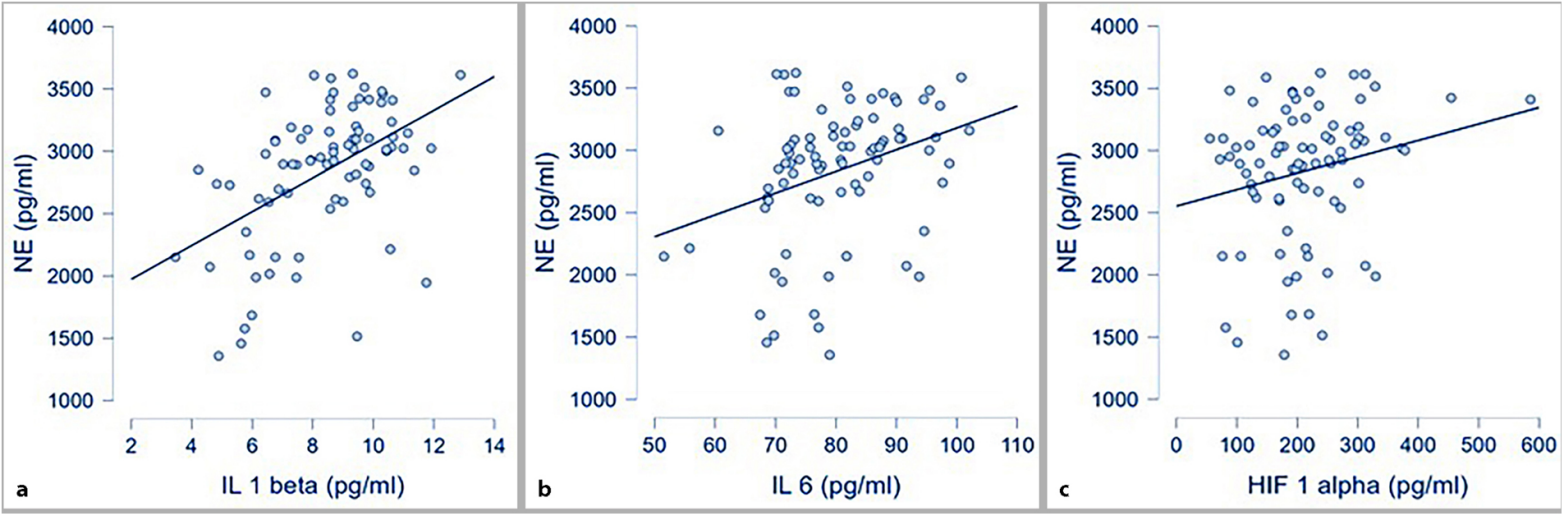
Correlations between inflammatory markers and NE; **(a)** IL 1 β and NE (*p* < .001; *r* 0.49); **(b)** IL 6 and NE (*p* = 0.003; *r* 0.32); **(c)** HIF 1α and NE (*p* 0.049; *r* 0.21).

The correlations between each inflammatory parameter and norepinephrine (NE) are shown in Figure 2. Statistically significant correlations were observed between IL-1β and NE, IL-6 and NE, as well as HIF-1α and NE.

**Figure 3:**
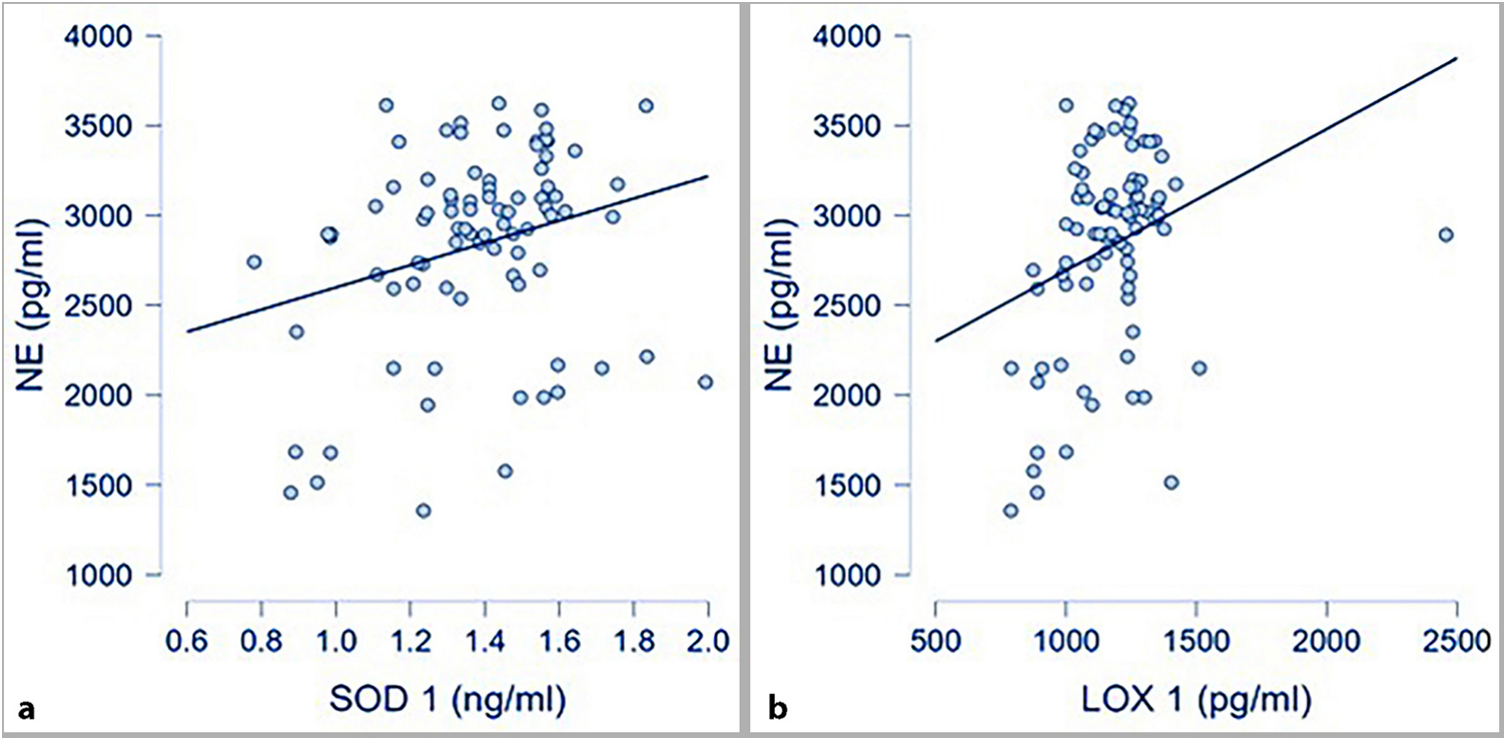
Oxidative stress markers and NE; **(a)** correlation between SOD-1 and NE (*p* 0.016; *r* 0.26); (b)* correlation between LOX-1 and NE (*p* .004; *r* 0.31; *spearman correlation for skewed distributed variables).

Circulating levels of oxidative stress markers (SOD-1 and LOX-1) were also correlated with circulating NE at a statistically significant level, as presented in Figure 3.

The analysis conducted to identify potential confounding factors found that none of the cardiovascular risk factors, including BMI, smoking, dyslipidemia, hypertension, and diabetes mellitus, was independently associated with NE levels, nor with the inflammatory markers (IL-1β, IL-6, HIF-1α) or oxidative stress indicators (SOD-1 and LOX-1) (see Supplementary Tables S1–S6 in the Supplementary Material).

## 4 Discussion

CAD is a disease state characterized by inflammation, increased oxidative stress and overstimulation of the SNS. Because these 3 aspects are interconnected, modulating any of them might prove useful for reducing the atherosclerotic burden and might, as well, have beneficial effects on the other components of this intricate network.

IL 1β plays a central role in local inflammation, being produced by monocytes/macrophages engulfed in the arterial wall in atherosclerosis. IL 1β mediates the production of IL 6, which in turn acts systemically activating more inflammatory cytokines and acute phase response proteins (13). Loss of viable myocardium in the setting of myocardial infarction is attributed to reperfusion injury and inflammatory response. Several recent trials have regarded pro-inflammatory cytokines as therapeutic targets, using specific antibodies in patients presenting with myocardial infarction. The largest of them, the CANTOS trial (Canakinumab Anti-inflammatory Thrombosis Outcome Study), which used Canakinumab, a monoclonal antibody binding selectively to IL 1β, enrolled patients with established CAD and residual inflammation. Its results proved a significant decrease of cardiovascular and cerebral events and cardiovascular death (14). However, the use of Tocilizumab, antibody targeting IL 6, did not show a significant clinical benefit in research conducted thus far (15, 16). An elevated pre-CABG surgery level of IL 6 has been associated with early graft occlusion and late cardiovascular events (17).

HIF 1 α is another molecule of interest, as it has links with both inflammation and oxidative stress. HIF 1 α stimulates macrophage activation, leading to lipid uptake and inflammation (including IL 1 β induction) and is also associated with reactive oxygen species (ROS) production by endothelial cells, activating NADPH oxidase genes (18). Also, HIF 1 α induces the expression of LOX 1 receptor at the cell surface, thus enhancing lipid uptake in macrophages (19). The HIF pathway remains of interest especially as its modulation could limit the effects of ischemia/reperfusion (for example in the of CABG surgery) or it could be used for ischemic pre-/post-conditioning, thus limiting the magnitude of an acute coronary event (20). However, recent research shows that maintaining an HIF 1 α intracellular homeostasis rather than totally inhibiting its effects could be the optimal approach to managing this important signaling pathway. HIF target-genes (such as adenosine receptors) could be activated directly. Some of these approaches may lead to novel pharmacologic strategies to prevent or treat organ injury in surgical patients (21). A recent trial showed that spironolactone is also involved in reducing HIF 1 α levels in patients undergoing CABG surgery (22).

SOD enzymes-SOD 1 being the major intracellular SOD- are part of the antioxidant defense system, implicated in lipid peroxidation and progression of atherosclerosis correlated with oxidative stress (23). However, in cases of increased SOD activity, such as in states of ischemia/reperfusion, the beneficial antioxidant effects are lost, increased damaged to the cell ensues (6, 8). CABG surgery induces increased levels of oxidative stress; hence the SOD system might be an efficient antioxidant (24).

Furthermore, increased ROS production also leads to the oxidation of native LDL to oxidized LDL (ox-LDL), which contributes to atherosclerotic plaque formation and progression by exerting its effects through its receptor-LOX-1 (25). LOX-1 expression is induced by several molecules: inflammatory cytokines (such as IL-1 and IL-6), angiotensin II, sheer stress, and glycation end-products. In turn, LOX-1 amplifies ROS formation by increasing NADPH oxidase activity. LOX-1 levels are upregulated in the heart after an ischemia/reperfusion event (CABG surgery), making it a potential target for therapy (26, 27).

Our study used a unique combination of biomarkers of inflammation (IL 1 beta, Il 6 and HIF 1 alpha) and oxidative stress (SOD 1 and LOX 1), each studied parameter proving to be correlated with increased levels of NE, neurohormone used to assess the systemic activity of the SNS.

To our knowledge, this is the first study to evaluate this combination of serum markers in patients with severe coronary artery disease (CAD) scheduled for surgical revascularization—a procedure that exposes patients to significant ischemia/reperfusion. The study demonstrates a statistical correlation between these markers and plasma norepinephrine (NE) levels, which serve as a surrogate for sympathetic nervous system (SNS) activation.

Recent research has proven that SNS is intimately associated with inflammation and oxidative stress (28, 29). Stimulation of central NE release also increases peripheral plasma NE: experimental studies conducted so far have shown that IL-1β and IL-6 injections determine short-lasting increases of NE (30, 31). In contrast, our study found high NE plasma concentrations correlated with high systemic levels of IL-1 β and IL-6, most likely a chronic state in patients with severe CAD.

Autonomic dysregulation is present throughout cardiovascular disease, with the interest in neuromodulation remaining increased. Previous research has proven that remodeling of the autonomic nervous system and the intrinsic cardiac nervous system occurs in various disease states, including the setting of myocardial infarction (32). NE exerts its effects on α and β adrenoceptors. α1 blockers exert anti-atherosclerotic effects by lowering blood pressure and modifying lipid profile (33). α2 adrenoceptors agonists also hold anti-atherosclerotic properties, possibly by reducing inflammation and stimulating oxidized LDL clearance, as recent animal studies show (34). Beta (β1, β2 and β3) blockers inhibit atherosclerosis progression by reducing circulating levels of proinflammatory cytokines, inhibiting oxidative stress, preventing LDL oxidation, decreasing monocyte adhesion to endothelium, and macrophage content in the atheroma plaque, conclusions drawn from experimental studies (35–37). However, the consequences of blunting the effect of SNS by renal denervation on atherosclerosis require further research, as present studies demonstrated that the procedure actually haslimiting adverse effects (38).

Recent research has proven that ischemic cardiomyopathy is accompanied by remodeling of the intrinsic cardiac nervous system, stellate ganglia, and higher nervous centers (39). Alterations of neurohumoral control and circulating catecholamines also occur (40). There is increasing evidence of the crosstalk between the immune system and the SNS (41). Cytokines stimulate the activation of SNS, which is further integrated at the hypothalamic level. In turn, NE can modulate the functions of the immune system.

Various markers of inflammation and oxidative stress work as a network and it is difficult to make associations between only one or few of them with cardiovascular risk and outcomes.

Moreover, excessive release of ROS can lead to cellular damage, negatively impacting not only the cardiovascular system, but also other bodily systems. Recent evidence suggests that oxidative stress plays a role in various neurodegenerative and neuropsychiatric disorders (42, 43).

## 5 Future directions

The prognostic value of a polyparametric approach was demonstrated in the setting of acute coronary syndromes, with the development of an Inflammatory Score, which unfortunately has not been further evaluated in other populations (44). It might be of interest to evaluate the prognostic value of a scoring system (including pro inflammatory markers, oxidative stress markers and NE) in the setting of ischemia/reperfusion injury (such as that induced by CABG). Furthermore, the use of targeted single cell treatments might be of interest in the management of selected patients (45).

## 6 Study limitations

Several limitations of the study must be mentioned. Firstly, the cytokines and oxidative stress markers that were tested are only a few of the molecules involved in the atherosclerotic process. Secondly, we only measured their circulating levels at one-point, serial determinations might be more suitable for establishing solid correlations.

## 7 Conclusion

In patients with CAD referred for CABG, SNS activation, as indicated by plasma NE levels, was correlated with disease severity, assessed by the SYNTAX I score. Additionally, NE levels were associated with markers of inflammation and oxidative stress, suggesting that inflammation, oxidative stress, and SNS activation form an interconnected network, where each component influences the others. It may be valuable to develop a scoring system that incorporates inflammation and oxidative stress markers to better identify patients who require a more aggressive strategy to reduce inflammation, oxidative stress, and modulate sympathetic nervous system (SNS) activity. Such a system could be particularly useful in the context of scheduled interventions, such as coronary artery bypass grafting (CABG) surgery, to tailor treatment and improve outcomes.

## Data Availability

The raw data supporting the conclusions of this article will be made available by the authors, without undue reservation.
