# Supplementary material for: The impact of inflammatory and oxidative stress biomarkers on the sympathetic nervous system in severe coronary atherosclerosis
Source: Front Cardiovasc Med. 2024 Oct 14;11:1480925. doi: 10.3389/fcvm.2024.1480925 (PMC11513282; doi:10.3389/fcvm.2024.1480925)
Supplement: Supplementary file 1 [file Table1.pdf]

SUPPLEMENTARY MATERIAL

Supplementary Table 1: Correlations between independent cardiovascular risk factors and NE

Pearson's Correlations

Variable		NE (pg. / ml)	Diabetes (0-no;1-yes)	Hypertension (0-no;1-yes)	Smoking status (0-no;1-yes)	Dyslipidemia (0-no;1-yes)	BMI (kg/m2)
1. NE (pg./ml)	Pearson's r	—					
	p-value	—					
2. Diabetes (0-no;1-yes)	Pearson's r	0.016	—				
	p-value	0.887	—				
3. Hypertension (0-no;1-yes)	Pearson's r	-0.044	0.202	—			
	p-value	0.694	0.065	—			
4. Smoking status (0-no;1-yes)	Pearson's r	0.071	0.141	-0.124	—		
	p-value	0.519	0.201	0.262	—		
5. Dyslipidemia (0-no;1-yes)	Pearson's r	0.047	-0.049	0.180	0.113	—	
	p-value	0.672	0.658	0.101	0.308	—	
6. BMI (kg/m2)	Pearson's r	0.050	0.023	0.057	-0.113	0.099	—
	p-value	0.648	0.832	0.607	0.305	0.369	—

* p < .05, ** p < .01, *** p < .001

Supplementary Table 2: The correlation between IL 1 beta and independent cardiovascular risk factors

IL 1 beta

Correlation Table

Variable		IL 1 beta (pg. /ml)	BMI (kg/m2)	Dyslipidemia (0-no;1-yes)	Smoking status (0-no;1-yes)	Hypertension (0-no;1-yes)	Diabetes (0-no;1-yes)
1. IL 1 beta (pg./ml)	Pearson's r	—					
	p-value	—					
	Spearman's rho	—					
	p-value	—					
2. BMI (kg/m2)	Pearson's r	-0.062	—				
	p-value	0.575	—				
	Spearman's rho	-0.069	—				
	p-value	0.530	—				
3. Dyslipidemia (0-no;1-yes)	Pearson's r	-0.026	0.099	—			
	p-value	0.814	0.369	—			
	Spearman's rho	-0.071	0.118	—			
	p-value	0.518	0.287	—			
4. Smoking status (0-no;1-yes)	Pearson's r	0.173	-0.113	0.113	—		
	p-value	0.115	0.305	0.308	—		
	Spearman's rho	0.156	-0.075	0.113	—		
	p-value	0.157	0.497	0.308	—		
5. Hypertension (0-no;1-yes)	Pearson's r	-0.045	0.057	0.180	-0.124	—	
	p-value	0.687	0.607	0.101	0.262	—	
	Spearman's rho	-0.034	0.072	0.180	-0.124	—	
	p-value	0.757	0.518	0.101	0.262	—	
6. Diabetes (0-no;1-yes)	Pearson's r	0.101	0.023	-0.049	0.141	0.202	—
	p-value	0.358	0.832	0.658	0.201	0.065	—
	Spearman's rho	0.100	0.034	-0.049	0.141	0.202	—
	p-value	0.365	0.758	0.658	0.201	0.065	—

* p < .05, ** p < .01, *** p < .001

Supplementary Table 3: IL 6 and independent cardiovascular risk factors

IL 6

Correlation Table

Variable		IL 6 (pg. /ml)	BMI (kg/m2)	Dyslipidemia (0-no;1-yes)	Smoking status (0-no;1-yes)	Hypertension (0-no;1-yes)	Diabetes (0-no;1-yes)
1. IL 6 (pg. /ml)	Pearson's r	—					
	p-value	—					
	Spearman's rho	—					
	p-value	—					
2. BMI (kg/m2)	Pearson's r	0.026	—				
	p-value	0.816	—				
	Spearman's rho	0.017	—				
	p-value	0.876	—				
3. Dyslipidemia (0-no;1-yes)	Pearson's r	-0.188	0.099	—			
	p-value	0.087	0.369	—			
	Spearman's rho	-0.165	0.118	—			
	p-value	0.134	0.287	—			
4. Smoking status (0-no;1-yes)	Pearson's r	-0.059	-0.113	0.113	—		
	p-value	0.593	0.305	0.308	—		
	Spearman's rho	-0.051	-0.075	0.113	—		
	p-value	0.647	0.497	0.308	—		
5. Hypertension (0-no;1-yes)	Pearson's r	-0.109	0.057	0.180	-0.124	—	
	p-value	0.324	0.607	0.101	0.262	—	
	Spearman's rho	-0.072	0.072	0.180	-0.124	—	
	p-value	0.518	0.518	0.101	0.262	—	
6. Diabetes (0-no;1-yes)	Pearson's r	-0.062	0.023	-0.049	0.141	0.202	—
	p-value	0.577	0.832	0.658	0.201	0.065	—
	Spearman's rho	-0.077	0.034	-0.049	0.141	0.202	—
	p-value	0.487	0.758	0.658	0.201	0.065	—

* p < .05, ** p < .01, *** p < .001

Supplementary Table 4: HIF 1 alpha and independent cardiovascular risk factors

HIF 1 alpha

Correlation Table

Variable		HIF 1 alpha (pg. /ml)	BMI (kg/m2)	Dyslipidemia (0-no;1-yes)	Smoking status (0-no;1-yes)	Hypertension (0-no;1-yes)	Diabetes (0-no;1-yes)
1. HIF 1 alpha (pg. /ml)	Pearson's r	—					
	p-value	—					
	Spearman's rho	—					
	p-value	—					
2. BMI (kg/m2)	Pearson's r	-0.091	—				
	p-value	0.411	—				
	Spearman's rho	-0.047	—				
	p-value	0.672	—				
3. Dyslipidemia (0-no;1-yes)	Pearson's r	0.145	0.099	—			
	p-value	0.188	0.369	—			
	Spearman's rho	0.163	0.118	—			
	p-value	0.140	0.287	—			
4. Smoking status (0-no;1-yes)	Pearson's r	0.079	-0.113	0.113	—		
	p-value	0.472	0.305	0.308	—		
	Spearman's rho	0.035	-0.075	0.113	—		
	p-value	0.753	0.497	0.308	—		
5. Hypertension (0-no;1-yes)	Pearson's r	0.170	0.057	0.180	-0.124	—	
	p-value	0.121	0.607	0.101	0.262	—	
	Spearman's rho	0.177	0.072	0.180	-0.124	—	
	p-value	0.106	0.518	0.101	0.262	—	
6. Diabetes (0-no;1-yes)	Pearson's r	-0.034	0.023	-0.049	0.141	0.202	—
	p-value	0.759	0.832	0.658	0.201	0.065	—
	Spearman's rho	-0.019	0.034	-0.049	0.141	0.202	—
	p-value	0.863	0.758	0.658	0.201	0.065	—

* p < .05, ** p < .01, *** p < .001

Supplementary Table 5: SOD-1 and independent cardiovascular risk factors

SOD 1

Correlation Table

Variable		SOD 1 (ng/ml)	BMI (kg/m2)	Dyslipidemia (0-no;1-yes)	Smoking status (0-no;1-yes)	Hypertension (0-no;1-yes)	Diabetes (0-no;1-yes)
1. SOD 1 (ng/ml)	Pearson's r	—					
	p-value	—					
	Spearman's rho	—					
	p-value	—					
2. BMI (kg/m2)	Pearson's r	0.109	—				
	p-value	0.325	—				
	Spearman's rho	0.126	—				
	p-value	0.254	—				
3. Dyslipidemia (0-no;1-yes)	Pearson's r	0.038	0.099	—			
	p-value	0.734	0.369	—			
	Spearman's rho	0.006	0.118	—			
	p-value	0.958	0.287	—			
4. Smoking status (0-no;1-yes)	Pearson's r	-0.039	-0.113	0.113	—		
	p-value	0.726	0.305	0.308	—		
	Spearman's rho	-0.040	-0.075	0.113	—		
	p-value	0.720	0.497	0.308	—		
5. Hypertension (0-no;1-yes)	Pearson's r	-0.117	0.057	0.180	-0.124	—	
	p-value	0.289	0.607	0.101	0.262	—	
	Spearman's rho	-0.143	0.072	0.180	-0.124	—	
	p-value	0.194	0.518	0.101	0.262	—	
6. Diabetes (0-no;1-yes)	Pearson's r	-0.106	0.023	-0.049	0.141	0.202	—
	p-value	0.338	0.832	0.658	0.201	0.065	—
	Spearman's rho	-0.101	0.034	-0.049	0.141	0.202	—
	p-value	0.363	0.758	0.658	0.201	0.065	—

* p < .05, ** p < .01, *** p < .001

Supplementary Table 6: LOX 1 and independent cardiovascular risk factors

LOX 1

Correlation Table

Variable		LOX 1 (pg. /ml)	BMI (kg/m2)	Dyslipidemia (0-no;1-yes)	Smoking status (0-no;1-yes)	Hypertension (0-no;1-yes)	Diabetes (0-no;1-yes)
1. LOX 1 (pg. /ml)	Pearson's r	—					
	p-value	—					
	Spearman's rho	—					
	p-value	—					
2. BMI (kg/m2)	Pearson's r	0.086	—				
	p-value	0.437	—				
	Spearman's rho	-0.094	—				
	p-value	0.395	—				
3. Dyslipidemia (0-no;1-yes)	Pearson's r	0.085	0.099	—			
	p-value	0.441	0.369	—			
	Spearman's rho	0.065	0.118	—			
	p-value	0.560	0.287	—			
4. Smoking status (0-no;1-yes)	Pearson's r	-0.080	-0.113	0.113	—		
	p-value	0.470	0.305	0.308	—		
	Spearman's rho	-0.065	-0.075	0.113	—		
	p-value	0.555	0.497	0.308	—		
5. Hypertension (0-no;1-yes)	Pearson's r	0.101	0.057	0.180	-0.124	—	
	p-value	0.363	0.607	0.101	0.262	—	
	Spearman's rho	0.115	0.072	0.180	-0.124	—	
	p-value	0.297	0.518	0.101	0.262	—	
6. Diabetes (0-no;1-yes)	Pearson's r	-0.099	0.023	-0.049	0.141	0.202	—
	p-value	0.372	0.832	0.658	0.201	0.065	—
	Spearman's rho	-0.091	0.034	-0.049	0.141	0.202	—
	p-value	0.408	0.758	0.658	0.201	0.065	—

* p < .05, ** p < .01, *** p < .001
